# The Effect of Bed Rest and Hypoxic Environment on Postural Balance and Trunk Automatic (Re)Actions in Young Healthy Males

**DOI:** 10.3389/fphys.2018.00027

**Published:** 2018-01-25

**Authors:** Nejc Šarabon, Igor B. Mekjavić, Ola Eiken, Jan Babič

**Affiliations:** ^1^Faculty of Health Sciences, University of Primorska, Koper, Slovenia; ^2^Laboratory for Motor Control and Motor Behaviour, S2P, Science to Practice, Ltd., Ljubljana, Slovenia; ^3^Department for Automation, Biocybernetics and Robotics, Jožef Stefan Institute, Ljubljana, Slovenia; ^4^Department of Biomedical Physiology and Kinesiology, Simon Fraser University, Burnaby, BC, Canada; ^5^Department of Environmental Physiology, Swedish Aerospace Physiology Centre, Royal Institute of Technology, Stockholm, Sweden

**Keywords:** inactivity, bed rest, hypoxia, balance, trunk, function

## Abstract

Prolonged inactivity, such as bed rest induces several detrimental changes within a short timeframe. Impaired postural balance and responses of trunk muscles to (un)expected perturbations were both shown to be impaired after bed rest. Certain populations (e.g., astronauts) are exposed to hypoxic environment in addition to inactivity, similar to bed rest. While the isolated negative effects of hypoxia on postural balance have been observed before, no study to date has examined the combined effects of hypoxia and bed rest on postural balance or trunk muscle responses. In this study, we examined the effects of 21-day exposure to three conditions: (i) bed rest in hypoxic environment (HBR), (ii) bed rest in normoxic environment (NBR), and (iii) ambulatory hypoxic environment (HAMB). Fourteen healthy male subjects crossed over between conditions in a randomized order, with a 4-month break between conditions to ensure full recovery. Most body sway parameters indicated a similar deterioration of postural balance following both HBR and NBR. Similarly, both anticipatory and reactive responses of the trunk muscles (m. erector spinae and m. multifidus) were impaired after HBR and NBR to a similar degree and mostly unchanged after HAMB. Certain body sway parameters were impaired after HAMB, confirming that hypoxia alone can undermine postural balance. On the other hand, some trunk responses were improved after HAMB. In conclusion, the results of our study confirmed previous findings on negative effects of bed rest, but showed little or no additional effect of hypoxia during bed rest. Physical activity during bed rest is encouraged to preserve neuromuscular functions of the trunk. While the HBR condition in our study resembled conditions during space missions, our results could be relevant to other populations, such as patients with pulmonary diseases exposed to bed rest.

## Introduction

Prolonged bed rest has been shown to induce several physiological and morphological changes, such as muscle atrophy, decrease in bone mineral density (Parry and Puthucheary, 2015) and impaired cognitive abilities (Lipnicki et al., 2009). Moreover, several studies have demonstrated a debilitation of neuromuscular function following bed rest. Yamanaka and colleagues (Yamanaka et al., 1999) reported an inhibition of H-reflex, but no change in motor evoked potentials after a 20-day bed rest, suggesting that the neuromuscular pathways most affected were the sensory ones. The inactivity/unloading effect of bed rest on the muscle sensory nerves may also be implicated in the delayed postural reflex (i.e., reactive) responses of trunk muscles to sudden mechanical perturbation following bed rest (Panjabi, 1992). Trunk stability functions play a role in preventing spinal injuries and allow for good quality of limb movements (Hoffman and Gabel, 2013). In aging adults confined to bed rest, muscle atrophy is more pronounced in antigravity muscles, including several trunk muscles (Ikezoe et al., 2012). However, bed rest can impair reflex pathways even in limb muscles such as *m. biceps brachii* (Nakazawa et al., 1997). To our knowledge, the only study that investigated the changes of trunk neuromuscular stabilizing functions following bed rest, found both anticipatory postural adjustments and postural reflex responses of trunk muscles to be impaired, with anticipatory postural adjustments nearly returning to baseline after 14-day rehabilitation period but postural reflex responses remaining substantially deteriorated even after such rehabilitation (Sarabon and Rosker, 2015).

Additionally, several studies have explored the change in postural balance after bed rest. Even a short 5-day bed rest protocol was found to impair postural stability, particularly when the stance task was accompanied with a dynamic head tilting (Mulder et al., 2014). Another study (Kouzaki et al., 2007) found a significant increase in postural sway during quiet stance after a 20-day bed rest; the balance decay taking place regardless of whether the bed rest included a resistance training countermeasure, or not. Furthermore, a 60-day bed rest was shown to impair both static (quiet stance on solid surface) as well as dynamic (standing on an unstable surface) balance in women. In dynamic balance, the anterior-posterior sway was increased more than the medial-lateral sway, suggesting a more pronounced effect of bed rest on distal muscles (Viguier et al., 2009). Interestingly, the static balance measures were restored faster in the group that underwent strength sessions and supine treadmill exercise within lower-body negative pressure box during bed rest, but no difference in the speed of recovery was reported for the dynamic balance measures between this exercise countermeasure group and a control group (no countermeasure). The authors suggested that this observation could have been a result of a specific effect that their intervention had on slow, oxidative muscle fibers, which are responsible for static balance (while faster anaerobic fibers are involved in dynamic balance). Since hypoxia disturbs the oxygen transportation to the muscles, it would be particularly interesting to see, whether it can further aggravate the static balance impairments, caused by bed rest.

Exposure to hypoxia affects postural stability (Fraser et al., 1987), even at relatively low altitudes (~2,500 m) (Wagner et al., 2011). Body sway changes following hypoxia seem to be more pronounced in the sagittal plane with eyes open (Nordahl et al., 1998). The present study was conducted within the framework of a larger study investigating the separate and combined effects of bed rest and hypoxia on the structure and function of several physiological system (PlanHab study; MEDS-IMPS and I.d.M.e.P.S, 2010; Debevec et al., 2014; Sundblad and Orlov, 2014). Specifically, the study was designed to address the concern that the hypoxic environments anticipated in future habitats on the Moon and Mars (Bodkin et al., 2006) may enhance the effect of reduced loading of the weight-bearing limbs due to the reduced gravity. Reports from the PlanHab study indicated that hypoxia augments the reduction in peak oxygen uptake induced by bed rest (Keramidas et al., 2016), but seems to have no effect on the whole body mass and fat-free mass reductions (Debevec et al., 2014). The focus of the present study was on the combined effect of bed rest and hypoxia on neuromuscular stabilizing functions of the trunk or postural balance control. Our hypothesis was that the changes in postural responses of the trunk muscles and static and dynamic balance will be different following separate and combined exposure to prolonged inactivity/unloading (bed rest) and normobaric hypoxia.

## Methods

### Participants

Fourteen healthy men (age: 26 ± 5 years, body height: 1.80 ± 0.05 cm, body weight: 76.9 ± 10.8) participated in the study. Each participant underwent careful medical examination prior to enrolment. The exclusion criteria were: history of deep vein thrombosis/pulmonary embolism, active malignancy, uncontrolled hypertension, history of cardiovascular disease, significant hepatic or renal disease, diabetes, chronic inflammatory disease, any significant impairment of the locomotor system, and vestibular or uncorrected visual disturbance. Each participant signed a written informed consent after being thoroughly informed of the study protocol and potential risks. The study protocol was confirmed by the Slovenian National Committee for Medical Ethics.

### Study design

We used a randomized crossover repeated measures design in this study. The subjects participated in three interventions, with the order of these interventions randomized: (i) bed rest in hypoxic environment (HBR), (ii) bed rest in normoxic environment (NBR), and (iii) ambulatory in hypoxic environment (HAMB). The trials were conducted at the Olympic Sport Centre Planica (Rateče, Slovenia), with the subjects being confined to one floor of this facility for the duration of each trial. In the HBR and HAMB conditions, hypoxia was established and maintained by a Vacuum Pressure Swing Adsorption System (b-Cat, Tiel, The Netherlands). The system maintained a level of hypoxia equivalent to that at an altitude of ~4,000 m, accounting for the fact that the facility is situated at 940 m. The partial pressure of inspired oxygen was 90.0 ± 0.4 mmHg in the HAMB and HBR trials, and 133.1 ± 0.3 mmHg in the NBR trials. Since this was a cross-over designed study, the interval between the interventions was a minimum of 3 months to ensure full recovery of the subjects prior to participating in the next intervention. For each trial, subjects arrived at the facility 5 days prior to the onset of the 21-d bed rest, and remained at the facility for 4 days after the conclusion of the bed rest. Baseline measurements before each of the intervention periods were performed during this 4-day period. In the HAMB trial, subjects were confined to the hypoxic facility as in the HBR and NBR trials, with the exception that they were requested to conduct daily 1-h sessions of supervised activity. The activity was designed to mimic the normal daily activity levels of the subjects. During the activity, the subjects' heart rate was monitored with the finger pulse oximeters, and the target heart rate during the activities was 50% of their hypoxic maximum heart rate. The type of exercise was alternated between cycle ergometry, and aerobics/dancing. In addition, during the HAMB trial, subjects were requested to maintain either a seated or standing position throughout the day. Laying supine on the bed was not permitted. At all times, they had to have their feet on the ground. To simulate upright standing activities, subjects were provided with other activities such as table football and darts. Thus, the main difference between the HBR and HAMB trials was the level of activity and loading on the weight-bearing limbs. This was zero in the HBR condition, and normal in the HAMB condition.

By and large, the bed rest protocol was conducted according to the guidelines of the European Space Agency (Heer et al., 2009; MEDS-IMPS and I.d.M.e.P.S, 2010; Sundblad and Orlov, 2014). In the bed rest conditions, participants were confined to bed for 21 days. All activities, including eating, showering, hygiene, etc. were conducted in the horizontal position. The subjects were monitored 24/7 during the bed rest trials, and CCTV cameras ensured compliance with the requirement of the bed rest protocol.

### Measurements

The measurement session included a short-standardized warm-up, static balance assessment and assessment of the stabilizing (re)actions of the trunk. The warm-up consisted of spot running with high knees for 2.5 min, 10 squats and 10 push-ups with the subject leaning to the wall at a ~30° angle and hands supported on the wall.

For static balance assessment, the participant was instructed to stand on a force platform (9260AA, Kistler, Winterthur, Switzerland) as still as possible. For each condition, three 30 s trails were performed. The conditions were: parallel stance with eyes open, parallel stance with eyes closed, and semi-tandem stance with eyes open (semi-tandem). The participants were barefoot for all trials and were requested to place their hands on their hips. Three components of the ground reaction force (vertical, anterior-posterior and medial-lateral) were measured using the force plate. The signals were sampled at 1,000 Hz and stored on a personal computer. Average center-of-pressure velocities, amplitudes and frequencies in anterior-posterior and medial-lateral directions were calculated and stored for further analysis.

Balance assessment was followed by evaluation of trunk stabilizing functions that included ant anticipatory postural adjustments and postural reflex responses measurements. EMG activity was recorded via self-adhesive pairs of electrodes (Blue Sensor N, Ambu A/S, Ballerup, Denmark) placed on designated muscles with 2 cm center-to-center distance, following the SENIAM recommendations (Hermens et al., 2000). The EMG activity of m. multifidus at L5 level m. erector spinae at L1 level and m. deltoideus anterior on the right side of the body was recorded. A reference electrode was placed on the right greater trochanter.

Measurements of anticipatory postural adjustments were performed on random visual cue (LED light) presented at random intervals. The participant stood at hips width, with his arms extended down by his sides, holding a 1.2 kg accelerometer bar with palms facing down. Upon the visual signal, the task was to raise the bar as fast as possible with extended arms from neutral position (i.e., the bar resting on hips) up to the shoulder height and return the bar back down slowly. For postural reflex responses measurements, participants stood relaxed, with their elbows flexed to 90° and palms slightly touching the handle of the weight, which was set at 8% of the individual's body mass. After the load release, the participants' task was to return the bar to the initial position, as quickly as possible. For both measurements, all trials were triggered in random manner every 5–12 s. Reliability of the applied test procedures for CoP sway during quiet stance tasks and trunk muscles' stability (re)actions has been tested in previous studies (Markovic et al., 2014; Voglar and Sarabon, 2014a,b; Sarabon and Rosker, 2015; Voglar et al., 2016) and we adopted the suggested reliability optimization guidelines in this study.

The EMG signals were amplified with a factor of 3,000 (Biovision, Wehrheim, Germany), A/D converted, and sampled at 10,000 Hz (USB-6343, National Instruments, Texas, USA). The main outcome parameters were average latencies and amplitudes of the responses, along with the rate of EMG rise in the first 50 ms of the response.

In addition to baseline and post-intervention measurements, daily measurements of capillary oxyhemoglobin saturation were conducted at 7:30 a.m. with a pulse oximeter.

### Statistical analysis

SPSS 20.0 software (SPSS Inc., Chicago, USA) was used for all statistical analyses. Descriptive statistics was calculated and reported as mean with entitled 95% confidence intervals. The Shapiro–Wilk test was used to test for normality of the distribution. Two-way repeated measures ANOVA [time (2) × condition (3)] was used to test the difference between the sessions. Greenhouse-Geiser correction was applied when Mauchly's sphericity test was statistically significant. Two-tailed *t*-tests with Bonferroni corrections were used for pairwise comparisons. For all the analyses, the level of statistical significance was set at *p* < 0.05.

## Results

During the NBR trial, capillary oxyhemoglobin saturation was 98 ± 1% prior to, and throughout the entire period of the bed rest intervention. In contrast, during HBR, capillary oxyhemoglobin saturation decreased from 97 ± 1% prior to the intervention to 83 ± 3% on the first day of the bed rest. Thereafter capillary oxyhemoglobin saturation gradually increased, attaining 98 ± 2% on the last (21st) day of the HBR. A similar response was observed during the HAMB trials, where capillary oxyhemoglobin saturation decreased from 97 ± 1% 1 day prior to the intervention, to 86 ± 1% on the first day of HAMB. As in the HBR condition, capillary oxyhemoglobin saturation gradually recovered, attaining a capillary oxyhemoglobin saturation value of 89 ± 3% on the last day of the 21-day intervention.

**Figure 1:**
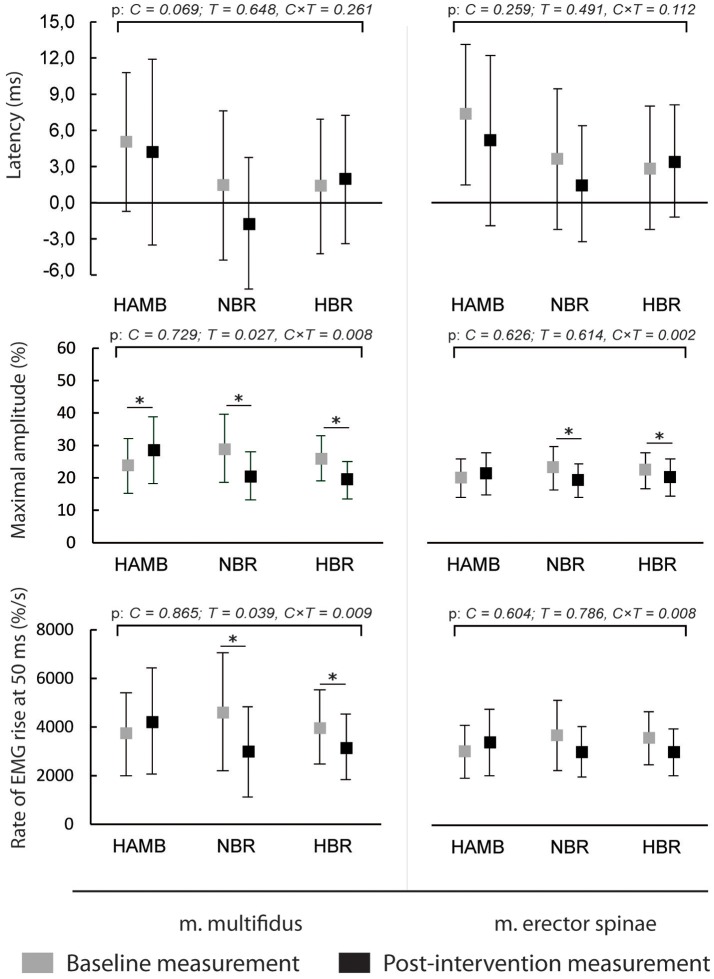
Differences in anticipatory postural adjustments' latencies, maximal amplitudes and rate of EMG rise before and after interventions (means and 95% CI).

**Figure 2:**
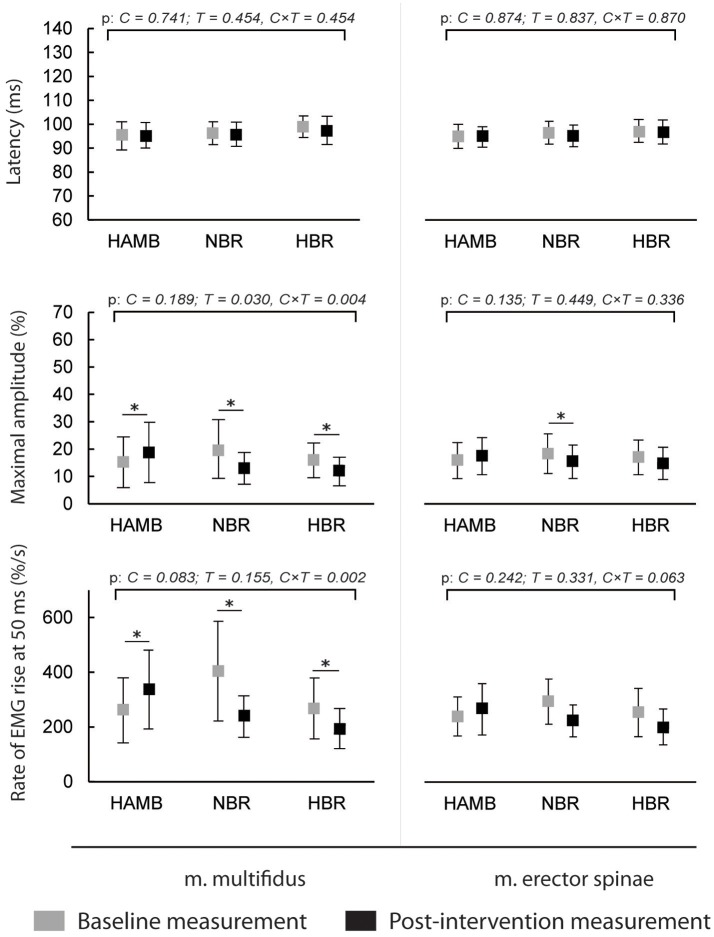
Differences in postural reflex responses' latencies, maximal amplitudes and rate of EMG rise before and after interventions (means and 95% CI).

The latencies of anticipatory postural adjustments ranged from −19.61 to 24.65 ms for m. multifidus and from −25.30 to 27.75 ms for ES, while postural reflex responses latencies ranged from 106 to 1.384 ms for m. multifidus and from 109 to 1.356 for m. erector spinae. No effect of condition, time or interaction was present for latencies (all *p* > 0.05). Latencies are presented in Figures 1, 2, respectively, along with the maximal amplitudes of the responses and the rate of EMG rise.

A decrease in anticipatory postural adjustments' maximal amplitudes was observed after HBR and NBR for both muscles, while m. multifidus exhibited increased amplitude after HAMB. Statistically significant interaction was present for both muscles [*F*_(2)_ = 6.671 and 9.347; *p* = 0.008 and 0.002; ES = 0.455 and 0.539 for m. multifidus and m. erector spinae, respectively]. However, there were no differences in post-scores between NBR and HBR for neither m. multifidus [*t*_(10)_ = 0.315; *p* = 0.760; ES = 0.010] nor m. erector spinae [*t*_(10)_ = −0.790; *p* = 0.448; ES = 0.059]. A decrease in rate of EMG rise for m. multifidus was observed after the NBR and HBR conditions [interaction: *F*_(2)_ = 6.369; *p* = 0.009; ES = 0.443] with no difference between the two [*t*_(10)_ = 0.142; *p* = 0.890; ES = 0.002]. There were no changes in rate of EMG rise for m. erector spinae between pre-post-scores, but there was a statistically significant interaction [*F*_(2)_ = 6.596; *p* = 0.063; ES = 0.292].

The amplitudes of postural reflex responses were increased after HAMB and decreased after NBR and HBR for m. multifidus [interaction: *F*_(2)_ = 7.879; *p* = 0.004; ES = 0.496]. There was no difference between NBR and HBR [*t*_(10)_ = 1.542; *p* = 0.154; ES = 0.192]. The trend was similar for m. erector spinae [interaction: *F*_(2)_ = 4.048; *p* = 0.038; ES = 0.336], however, a significant difference was present only after NBR. The rate of EMG rise changes for m. multifidus were the same as for the maximal amplitude—an increase was observed after HAMB and a decrease after NBR and HBR [interaction: *F*_(2)_ = 9.640; *p* = 0.002; ES = 0.546]. Again, no difference was shown between the bed rest conditions [*t*_(10)_ = −1.692; *p* = 0.122; ES = 0.223]. No changes in the rate of EMG rise were observed for m. erector spinae [interaction: *F*_(2)_ = 3.307; *p* = 0.063; ES = 0.292].

**Figure 3:**
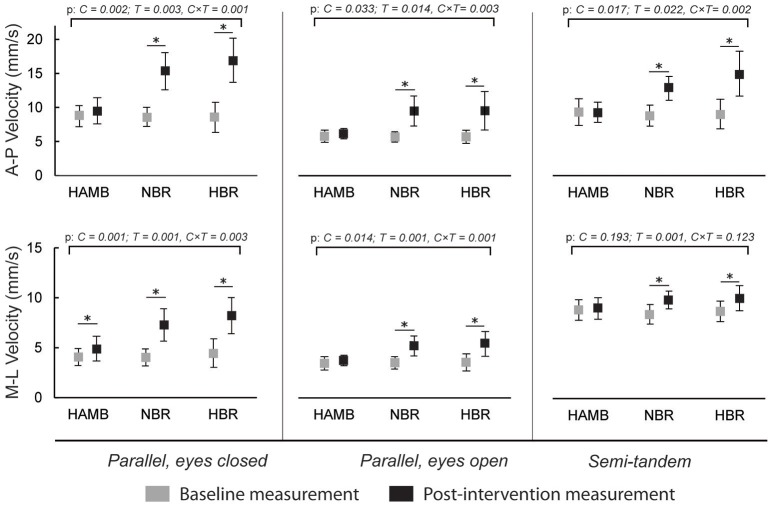
Differences in antero-posterior (A-P) and medio-lateral (M-L) body sway velocities in three different stances, before and after interventions (means and 95% CI).

Figure 3 presents the changes in antero-posterior and medio-lateral body sway velocity. An increase was present after NBR and HBR in both directions for all stances (*p* = 0.001 – 0.123 for interaction), and additionally for medio-lateral velocity after HAMB in parallel stance with eyes closed. However, this increase was significantly smaller than after NBR and HBR [*t*_(9 and 12)_ = −3.708 and −4.441; *p* = 0.005 and 0.001, ES = 0.604 and 0.642, respectively]. No differences were present between NBR and HBR for body sway velocity in any stances or directions [*t*_(12)_ = 1.812 – 0.597; *p* = 0.097 – 0.230; ES = 0.029 – 0.230].

A significant interaction was found for antero-posterior body sway amplitude in parallel stance with closed eyes [*F*_(2)_ = 4.983; *p* = 0.019; ES = 0.356] and parallel stance with eyes open [*F*_(2)_ = 4.435; *p* = 0.025; ES = 0.307]. In both stances, there was a statistically significant increase after NBR (*p* < 0.001) and HBR (*p* = 0.001 – 0.003), but not in HAMB. In semi-tandem stance, the increase was observed only after HBR [*t*_(13)_ = −2.525; *p* = 0.025; ES = 0.329], but the interaction effect was not statistically significant [*F*_(2)_ = 2.986; *p* = 0.073; ES = 0.230]. The medio-lateral body sway amplitude in semi-tandem stance increased only after HAMB [*t*_(11)_ = −2.447; *p* = 0.032; ES = 0.352], and there was no statistically significant interaction [*F*_(2)_ = 0.139; *p* = 0.871; ES = 0.014]. In parallel stance with closed eyes, the increases were observed after all conditions, but were more pronounced after NBR and HBR [interaction: *F*_(2)_ = 4.382; *p* = 0.028; ES = 0.327]. Higher medio-lateral amplitude values were also seen after NBR and HBR in parallel stance with eyes open, but there was no statistically significant interaction [*F*_(2)_ = 2.511; *p* = 0.106; ES = 0.201].

The mean antero-posterio frequency during parallel stance with closed eyes increased after NBR and HBR (interaction: *F* = 3.600; *p* = 0.048; ES = 0.286). In parallel stance with eyes open, the increase was observed after HAMB and HBR, but the interaction was not significant [*F*_(2)_ = 3.381; *p* = 0.054; ES = 0.253]. No differences were observed after any condition for mean antero-posterior frequency in semi-tandem stance or mean medio-lateral frequency in either of the parallel stances. In semi-tandem stance, the mean medio-lateral frequency increased after all conditions, with no statistically significant interaction [*F*_(2)_ = 0.756; *p* = 0.483; ES = 0.070].

## Discussion

This was the first study to analyze combined and isolated effects of bed rest and hypoxia on neuromuscular responses of the trunk musculature and on postural balance. As expected, NBR and HBR caused greater deteriorations in most of the analyzed measures than HAMB, however, statistically significant differences between NBR and HBR were observed only in a few measures. No changes were observed in anticipatory postural adjustments or postural reflex responses latencies in any of the conditions. The average amplitudes of both responses of m. multifidus were higher in the HAMB trial. The amplitudes of anticipatory postural adjustments were decreased for both muscles after NBR and HBR (with no differences between the two). The postural reflex responses amplitudes of m. multifidus were also lowered following both BR conditions, but only after NBR for m. erector spinae. Additionally, the rate of EMG rise for m. multifidus was decreased after NBR and HBR for both responses, with no changes observed for m. erector spinae. The above results did not show a potential of hypoxic environment to further augment the unfavorable effects bed rest has on trunk stabilizing functions, whereas the HAMB intervention (most probably its exercise content) even improved certain functions. Previous studies have demonstrated an acute decrease in maximal voluntary contraction force following hypoxia (most likely resulting from decreased activation of highly oxygen-dependent slow motor fibers) (Dousset et al., 2001a). An animal study (Dousset et al., 2001b), investigating neuromuscular responses after chronic exposure to hypoxia showed a decreased response of muscle afferents, while the nerve conduction velocity was higher, which could be the mechanism behind unchanged latencies in our study. However, it has further been demonstrated that both acute and chronic hypoxia have negative effects on muscle afferent activity in humans (Dousset et al., 2003). The improvements following HAMB in our study could have also occurred due to a learning effect, as pre- and post-intervention measurements within the conditions were conducted 25 days apart, while the interval between exposures to different conditions and corresponding measurements was a minimum of 4 months.

The body sway velocity in both directions was increased after NBR and HBR in all of the stances, but mostly remained the same after HAMB. Similarly, antero-posterior amplitude increased after NBR and HBR in both parallel stances. However, it was only increased following HBR in semi-tandem stance. The medio-lateral amplitude increased after NBR and HBR in parallel stance with eyes closed, after HAMB and HBR in parallel stance with eyes open and interestingly, only after HAMB in semi-tandem stance. Changes in Mean Frequency were also not uniform across stances and directions. The mean medio-lateral frequency was increased after all conditions in the semi-tandem stance, while the mean A-frequency was higher after NBR and HBR in parallel stance with eyes closed, and after HAMB and HBR in parallel stance with eyes open. The antero-posterior amplitude in semi-tandem stance was the only measure that HAMB condition seemed to further increase (more than the bed rest alone). Based on the data from previous studies (Nordahl et al., 1998), demonstrating hypoxia to have a greater effect on postural stability in the sagittal plane, we would expect such results across more than one measure. However, the majority of the postural stability measures deteriorated to the same degree after NBR and HBR. Moreover, changes in frontal plane following HAMB were just as frequent as those in sagittal plane. Our results confirm the findings of previous studies suggesting that hypoxia alone can impair certain measures of postural balance (Fraser et al., 1987; Nordahl et al., 1998; Wagner et al., 2011; Degache et al., 2012), but do not indicate that hypoxia deteriorates antero-posterior measures in particular, as previously reported (Nordahl et al., 1998). On the other hand, this study did not provide any clear evidence for hypoxia to augment the negative changes of prolonged inactivity. It is important to note that previous studies (Fraser et al., 1987; Nordahl et al., 1998; Wagner et al., 2011; Degache et al., 2012) mostly explored acute effects of hypoxia. In our study, the exposure time was of sufficient length for at least some short-term adaptations (Powell et al., 1998) to occur.

While the results of the present study are non-uniform and equivocal, they do support the notion that minimizing the period of inactivity in populations simultaneously exposed to hypoxia and physical inactivity should be the primary goal when trying to preserve neuromuscular function. With regards to astronauts on missions to the Moon and Mars, who may be exposed to the hypoxic conditions within the habitats, the current practice of daily exercise will minimize deterioration of neuromuscular function. Since the focus of the overall project was the effect of hypoxic inactivity/unloading on astronauts in future missions to the Moon and Mars, the current study was conducted on healthy young male subjects, but the results could be of relevance to specific patient populations rendered either inactive and/or hypoxic. Thus, preservation of neuromuscular function could be jeopardized in patients suffering chronic obstructive pulmonary disease or cardiac insufficiency.

## Ethics statement

This study was carried out in accordance with the recommendations of Republic of Slovenia National Medical Ethics Committee with written informed consent from all subjects. All subjects gave written informed consent in accordance with the Declaration of Helsinki. The protocol was approved by the Republic of Slovenia National Medical Ethics Committee (no. 205/02/11).

## Author contributions

NŠ, IM, OE, and JB designed the study and performed the experiments. NŠ analyzed the data. NŠ, IM, OE, and JB wrote the manuscript.

### Conflict of interest statement

NŠ was employed by company S2P, Science to Practice, Ltd. The other authors declare that the research was conducted in the absence of any commercial or financial relationships that could be construed as a potential conflict of interest.
